# P-1465. Real-World Treatment Patterns in Adult Patients Infected with Extra-Intestinal Pathogenic *Escherichia Coli* in the United States

**DOI:** 10.1093/ofid/ofae631.1637

**Published:** 2025-01-29

**Authors:** Brenda Rattanavong, Sanjukta Basu, Atara Laor, Liga Bennetts, Ziyan (Jenny) Wei, Antoine El Khoury, Jeroen Geurtsen, Nina Ahmad, Maureen P Neary

**Affiliations:** Amaris Consulting, Montreal, Quebec, Canada; Amaris Consulting, Montreal, Quebec, Canada; Amaris Consulting, Montreal, Quebec, Canada; Amaris Consulting, Montreal, Quebec, Canada; Amaris Consulting, Montreal, Quebec, Canada; Janssen Global Services LLC, Titusville, New Jersey; Janssen Vaccines & Prevention BV, Leiden, Netherlands; Janssen Global Services LLC, Titusville, New Jersey; Janssen Global Services, LLC, Raritan, New Jersey

## Abstract

**Background:**

Extraintestinal pathogenic *E. coli* (ExPEC) are often treated with multiple classes of antibiotics, however, real-world treatment patterns are poorly characterized. We sought to identify evidence about treatments used for adult patients infected with ExPEC in the US.

Figure 1

**Figure:**
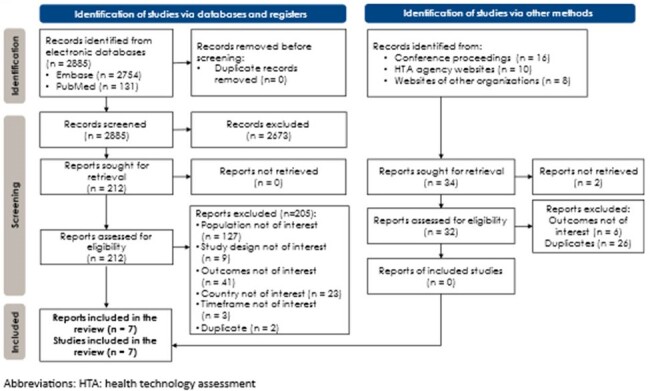

PRISMA flowchart

**Methods:**

A systematic literature review was conducted in EMBASE, MEDLINE, and MEDLINE In-Process databases to identify US studies of treatments used for ExPEC infections in real world settings, published Jan. 2010–Sept. 2023.

Figure 2

**Figure:**
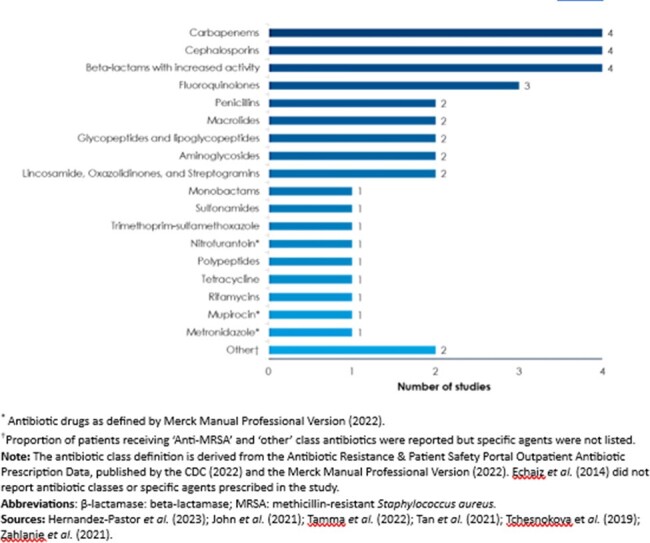

Antibiotic classes used for the treatment of ExPEC infections observed across studies

**Results:**

Seven publications met eligibility criteria for inclusion (**Fig 1**). Infections were usually community acquired (CA) (67.8% - 94.3%). Median treatment duration was reported in 3 studies and ranged 2.72 - 13 days. While various antibiotic classes used to treat ExPEC infections were reported, broad-spectrum antibiotics were used frequently (**Fig 2**). Patients with CA *E. coli* pneumonia and older patients (≥70 yrs.) with urinary tract infection commonly received fluoroquinolones (FQ) or cephalosporins. Carbapenem was frequently prescribed in patients with ceftriaxone-resistant *E. coli* and those with extended spectrum β-lactamase producing *E. coli* (ESBL-EC). Treatment patterns following an index visit were described in 3 studies; patients often received multiple antibiotics, one study reported that 82.8% of patients received >1 antibiotic course. Among patients with non-ESBL-EC and those with *E. coli* bacteremia, ceftriaxone was the most frequent antibiotic used for de-escalation of empiric piperacillin-tazobactam following susceptibility cultures. Adequate therapy (i.e. antibiotic/pathogen match) was reported to be more likely among patients with non-ESBL-EC compared with ESBL-EC and among patients admitted to hospitals during weekends, while inadequate therapy was more common in patients with *E. coli* ST131. One study reported that 39% of FQ-resistant patients were switched to adequate therapy.

**Conclusion:**

These findings highlight the complexity of managing ExPEC infections which involve a wide range of antibiotics and often > 1 antibiotic course contributing to the rise of antimicrobial resistance (AMR) with increased morbidity and mortality. There is an unmet need for more data on treatment patterns and associated AMR rates for specific real-world treatment patterns.

**Disclosures:**

**Brenda Rattanavong, PharmD**, Janssen Global Services, LLC: As a medical writer, I receive payment from the sponsor to support drafting of the abstract. **Sanjukta Basu, Ph.D**, Amaris Consulting: Advisor/Consultant **Atara Laor, BSc.**, Amaris Consulting: Advisor/Consultant **Liga Bennetts, PhD**, Amaris Consulting: Advisor/Consultant **Ziyan (Jenny) Wei, Msc**, Amaris Consulting: Advisor/Consultant **Antoine El Khoury, PhD**, Janssen Global Services: Advisor/Consultant|Janssen Global Services, LLC: Employee of Janssen Global Services, LLC **Jeroen Geurtsen, PhD**, Janssen Vaccines & Prevention BV: Advisor/Consultant|Janssen Vaccines & Prevention BV: Employee of Janssen Vaccines & Prevention BV **Nina Ahmad, MD**, Janssen Global Services, LLC: Employee of Janssen Global Services, LLC **Maureen P. Neary, PhD, MS**, Janssen Global Services, LLC: Employee of Janssen Global Services, LLC

